# Artificial intelligence and the future of radiographic scoring in rheumatoid arthritis: a viewpoint

**DOI:** 10.1186/s13075-022-02972-x

**Published:** 2022-12-12

**Authors:** Alix Bird, Lauren Oakden-Rayner, Christopher McMaster, Luke A. Smith, Minyan Zeng, Mihir D. Wechalekar, Shonket Ray, Susanna Proudman, Lyle J. Palmer

**Affiliations:** 1 Australian Institute of Machine Learning, University of Adelaide, Corner Frome Road and North Terrace, Adelaide, SA 5000 Australia; 2 School of Public Health, The University of Adelaide, North Terrace, Adelaide, SA 5000 Australia; 3 Department of Rheumatology, Austin Health, Heidelberg, VIC 3084 Australia; 4 Department of Rheumatology, Flinders Medical Centre, and College of Medicine and Public Health, Flinders University, Bedford Park, SA 5042 Australia; 5 Artificial Intelligence and Machine Learning, GlaxoSmithKline, South San Francisco, CA USA; 6 Department of Rheumatology, Royal Adelaide Hospital, Adelaide, SA 5000 Australia

**Keywords:** Rheumatoid arthritis, Radiographic scoring, Artificial intelligence, Deep learning

## Abstract

Rheumatoid arthritis is an autoimmune condition that predominantly affects the synovial joints, causing joint destruction, pain, and disability. Historically, the standard for measuring the long-term efficacy of disease-modifying antirheumatic drugs has been the assessment of plain radiographs with scoring techniques that quantify joint damage. However, with significant improvements in therapy, current radiographic scoring systems may no longer be fit for purpose for the milder spectrum of disease seen today. We argue that artificial intelligence is an apt solution to further improve upon radiographic scoring, as it can readily learn to recognize subtle patterns in imaging data to not only improve efficiency, but can also increase the sensitivity to variation in mild disease. Current work in the area demonstrates the feasibility of automating scoring but is yet to take full advantage of the strengths of artificial intelligence. By fully leveraging the power of artificial intelligence, faster and more sensitive scoring could enable the ongoing development of effective treatments for patients with rheumatoid arthritis.

## Introduction

Rheumatoid arthritis (RA) is a common polyarthritis that causes inflammation and destruction of synovial joints [1]. RA affects 0.5–1% of the global population and is associated with disability, work loss, and premature death [2]. In the USA, this equates to a cost of $19.3 billion annually in both healthcare and indirect expenses [3]. With advances in the use of disease-modifying antirheumatic drugs (DMARDs) and the advent of new biologic DMARDs (bDMARDs), the ability to suppress joint inflammation has improved considerably [4]. Clinical remission and the prevention of joint damage are now achievable for many patients [5]. Progress in developing new pharmacotherapies for RA has been enhanced by radiographic scoring systems that carefully quantify the severity and rate of progression of joint damage in clinical trials [6]. However, it has become increasingly difficult to verify the superiority or inferiority of new therapies with established radiographic scoring systems given their low sensitivity to the less severe joint damage which has become the clinical norm [7]. Furthermore, while desirable for use in routine clinical practice, current radiographic scoring systems are too time-intensive to be used in this context [8].

Deep learning is a type of artificial intelligence (AI) that offers a potential solution to the constraints of radiographic scoring, as it can efficiently and consistently identify patterns in imaging data. This article reviews the use of radiographic scoring in RA and explores the proposition that deep learning offers new opportunities to improve upon past scoring systems. We first discuss the history of radiographic scoring in RA and assess the current state-of-the-art in the application of deep learning to this task through a scoping review of the literature. We then look toward the future of deep learning in rheumatology and discuss how it may accelerate the search for new therapies and allow for the routine use of complex radiographic scoring in the clinical setting for the first time.

## Background

### Radiographic scoring

Plain radiography has been the standard imaging modality for the diagnosis and monitoring of joint damage in RA due to its ability to detect erosions (the pathological hallmark of the disease) and joint space narrowing [9], which are considered to be the most reliable features in determining progression of joint damage. In clinical trials, the reliability of a radiographic scoring system as a surrogate outcome is conditional on its ability to predict disability associated with RA. Scoring methods have been shown to correlate with the Health Assessment Questionnaire, a self-reported measure commonly used in RA to assess functional status and disability [10]. Consequently, the key assumption that underpins many RA drug trials is that prevention of joint destruction will improve functional outcomes.

Radiographic scoring has been evolving since its conception in the 1940s, to achieve a balance between sensitivity to change, interobserver agreement, and time taken for evaluation. The initial approach—the Steinbrocker method—assigned a single global score for the entire hand [11], but was inadequate for many trials as there was often extensive disease progression before the overall score increased [12]. Sharp et al. proposed a more complex method in 1971 that assessed individual joints for erosions and JSN, and included joints based on the reproducibility of their assessment, and on ensuring enough joints were included to be representative of disease [13]. The Larsen score, developed in 1977, included additional radiographic findings of soft tissue swelling and periarticular osteoporosis [14]. While these tend to occur earlier in disease and therefore may be more sensitive [14], these features are considered less reliable as they are dependent on radiographic technique and are subject to higher interobserver variability [9].

Today, the van der Heijde modification of the Sharp score (SvdH) is most commonly used in clinical trials. SvdH scoring was used in 73% of trials conducted between 1994 and 2020 [7]. The SvdH score was developed in 1989 and improves upon the original Sharp score by including the metatarsophalangeal joints and first interphalangeal joint of the feet and eliminating some joints of the wrist [15]. These modifications served to improve the sensitivity to change and interobserver agreement, as joints in the feet (especially the first metatarsophalangeal joint) are often affected early in RA, and a number of the joints in the wrists (such as the lunotriquetral joint and first interphalangeal joint) can be difficult to reliably assess as they can be obscured by overlapping surrounding structures.

Despite the extensive effort to improve upon the sensitivity, speed, and interobserver agreement of radiographic scoring, scoring systems in current use remain constrained by poor reproducibility and low sensitivity to change and by the prohibitive length of time taken for expert evaluation [16]. These constraints of manual scoring highlight the potential role of deep learning given that a trade-off in these qualities is inevitable when depending on human scorers. Deep learning is well positioned to further improve upon scoring, particularly by improving sensitivity to change while simultaneously increasing reproducibility and dramatically reducing the time required to score a radiograph.

### Deep learning

**Fig. 1:**
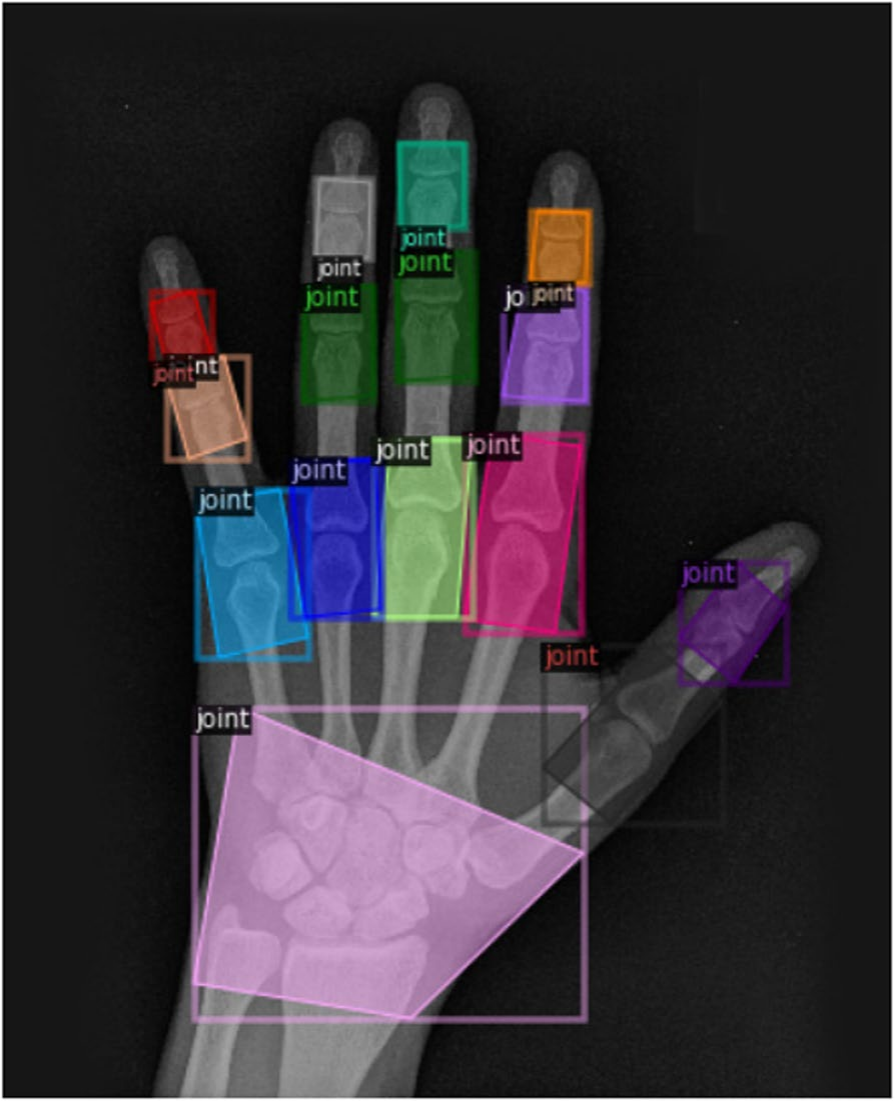
Output of neural network trained to detect joints in the hands

Deep learning is a type of AI that is currently the most powerful method in many applications, particularly for problems using image data. Deep learning models are able to train from a wide variety of input data, such as medical imaging or text from electronic health records (EHR). When dealing with imaging data, a common approach is to build these models using convolutional neural networks (CNNs), where inputs are passed through many layers that can identify increasingly abstract image features. Earlier layers may identify simple features such as edges and textures while later layers can identify more abstract concepts such as the presence or severity of a disease. The algorithm is updated with each example it processes to gradually become more accurate and can reach human-level performance across a variety of tasks [17]. Figure 1 shows an example of a model trained to identify the joints of a hand in a patient with RA.

In rheumatology more broadly, deep learning-based models are being developed to tackle a wide range of tasks such as automating testing for antinuclear antibodies [18], interpretation of synovial ultrasounds [19], and predicting diagnoses from an EHR [20]. Despite the promise of these techniques, many models have often failed to be implemented clinically, a phenomenon termed the “implementation gap” [21], which highlights the true complexity of applying such technologies in the clinical context. With many emerging applications, there is an increasing need for expert clinical guidance to develop algorithms that can better leverage the strengths of deep learning [22].

## Scoping review

In the past 4 years, deep learning has sparked a resurgence of research interest in automating measurement of articular changes, as it offers the prospect of a robust and fully automated approach. We conducted a scoping review of the literature to examine the progress in automating radiographic scoring, and the lessons to consider moving forward. The literature review was conducted according to the PRISMA guidelines for scoping reviews [23].

### Eligibility criteria

Publications were eligible if the study applied machine learning to the automation of radiographic scoring in RA. They were included if they met the following criteria: (1) models were applied to radiographic scoring of RA (any scoring system was accepted) and (2) the study used deep learning or machine learning-based algorithms. Studies were excluded if (1) they were using imaging modalities other than plain radiography, (2) they used non-AI methods, or (3) the study lacked sufficient information for analysis.

### Search strategies

Searches were conducted in four online databases—Embase, PubMed, Web of Science, and Scopus from inception to 24 January 2022.

Keywords were selected to search various databases based on consultations with an academic librarian. These included (1) “rheumatoid arthritis,” “inflammatory arthritis,” “rheumatism,” “arthritis,” “polyarthritis,” “rheumatic,” (2) “machine learning,” “deep learning,” “artificial intelligence,” “computer aided diagnosis,” “neural network,” “convolutional,” “decision tree,” “random forest,” “precision medicine,” and (3) “radiodiagnosis,” “radiograph,” “x ray,” and “imaging.” These searches were limited to studies published in English.

### Selection process

Study selection was conducted independently by two reviewers. Records were first independently screened by AB and LAS based on titles and abstracts. Records that initially met eligibility criteria were assessed using the full text. Discrepancies between the reviewers were resolved by consultation with a third reviewer (LOR).

### Data collection

As detailed in Table 1, data from the eight eligible studies from seven different authors were compiled. The data extracted included (1) specifics of the task at hand (what scoring method was used and which joints were assessed), (2) the size of the training data set, (3) the machine learning method employed, (4) the test dataset size, and (5) the performance reported. The reviewers recorded the sensitivity and specificity of each model if these could be derived from metrics reported.

**Table 1:** Performance of deep learning models to automate radiographic scoring in rheumatoid arthritis

Authors	Task	Training dataset	Test dataset and method	Deep learning method	Performance
Morita et al. 2017 [24] Full manuscript	1. Joint detection 2. SvdH erosion/JSN scores for MCPs and PIPs	45 radiographs	45 radiographs using leave-one-out cross-validation	HOG and SVM for joint detection and SVR to estimate the erosion and narrowing scores	Erosions 50.9% accuracy Absolute error 0.59 ± 0.24 JSN 64.3% accuracy Absolute error 0.43 ± 0.12
Morita et al. 2018 [25] Full manuscript	1. Joint detection 2. SvdH erosion/JSN scores for MCPs and PIPs	90 radiographs	90 radiographs using leave-one-out cross-validation	HOG and SVM for joint detection and ridge regression to estimate the erosion and narrowing scores	Erosions 53.3% accuracy Absolute error 0.63 ± 0.32 JSN 60.8% accuracy Absolute error 0.47 ± 0/13
S Murakami et al. 2018 [26] Full manuscript	1. Joint detection 2. Presence or absence of erosions	129 radiographs	30 radiographs, hold-out validation	MSGVF to identify regions of interest Three-layer CNN for erosion classification	Erosions Sensitivity = 0.805 Specificity = 0.9916
Rohrbach et al. 2019 [27] Full manuscript	1. Ratingen erosion scores for MCPs and PIPs	277 radiographs	31 radiographs, hold-out validation	VGG16 inspired model	Erosions Sensitivity = 0.924 Specificity = 0.758
Hirano et al. 2019 [28] Full manuscript	1. Joint detection 2. SvdH erosion/JSN scores for MCPs and PIPs	186 training radiographs from 108 patients	30 radiographs, hold-out validation	Uses a cascade classifier using Haar-like features to detect joints Then uses a CNN for the classification of erosions and JSN—two conv layers, two pooling, and three fully connected	Erosions Sensitivity = 0.424, 0.348 Specificity = 0.894, 0.882 JSN Sensitivity = 0.880, 0.942 Specificity = 0.748, 0.520
Deimel et al. 2020 [29] Abstract	1. Joint detection 2. SvdH JSN scores for MCPs and PIPs	5191 radiographs from 640 patients: 2207 train, 1150 validation	1834 radiographs, hold-out validation	ROI extraction with a deep learning model that considers appearance and spatial relationship in labeling	Calculated from the confusion matrix JSN MCPs Sensitivity = 0.844 Specificity = 0.909 JSN PIPs Sensitivity = 0.863 Specificity = 0.870
Huang et al. 2020 [30] Abstract	1. Joint detection 2. SvdH erosion/JSN scores for MCPs, PIPs, CMCs, and wrist	Approximately 960 hand radiographs from 309 patients diagnosed with RA	430 radiographs from 141 patients, hold-out validation	Deep adaptive graph	JSN Sensitivity = 0.808 Specificity = 0.919 Reported explicitly, but uses cutoff joint space score ≥ 2 Data not available to calculate sens/spec for JSN vs no JSN
Izumi et al. 2020 [31] Abstract	1. Joint detection 2. SvdH erosion scores for PIPs, IP and MCPs	104 x-rays	104 radiographs, 5-fold cross-validation	CNN	5-fold cross-validation Mean error of 0.412 per joint (of SvdH score) No further data available

*CMC*, carpometacarpal; *CNN*, convolutional neural network; *HOG*, histogram of gradients; *IP*, interphalangeal; *MCP*, metacarpophalangeal; *MSGVF*, multiscale gradient vector flow; *PIP*, proximal interphalangeal; *ROI*, region of interest; *SVM*, support vector machine; *SVR*, support vector regression; *VGG*, visual geometry graph

### Selection of studies

**Fig. 2:**
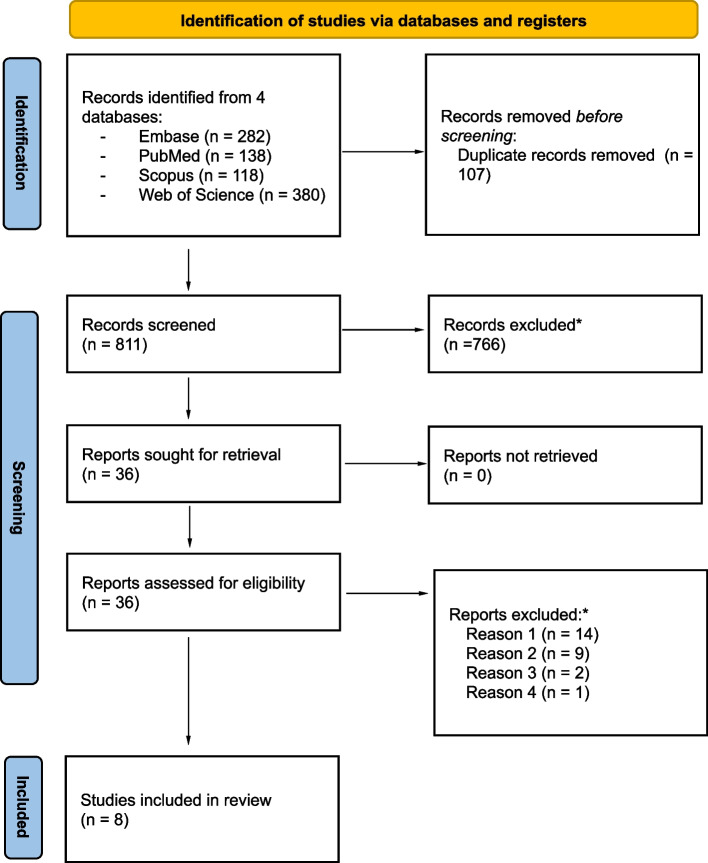
Flow diagram regarding study identification and selection [32]. *Reason 1: not investigating radiographic scoring; reason 2: not using machine learning; reason 3: using a different imaging modality; reason 4: lacked sufficient information for analysis

After duplicates were removed, 811 titles and abstracts were reviewed independently by two reviewers. Of these, 766 were excluded and the remaining 36 records were assessed using the full text of the publication. Following this, 5 studies and 3 abstracts were found to meet the criteria and were included in the scoping review. See the flow diagram in Fig. 2.

### Characteristics of included studies

**Fig. 3:**
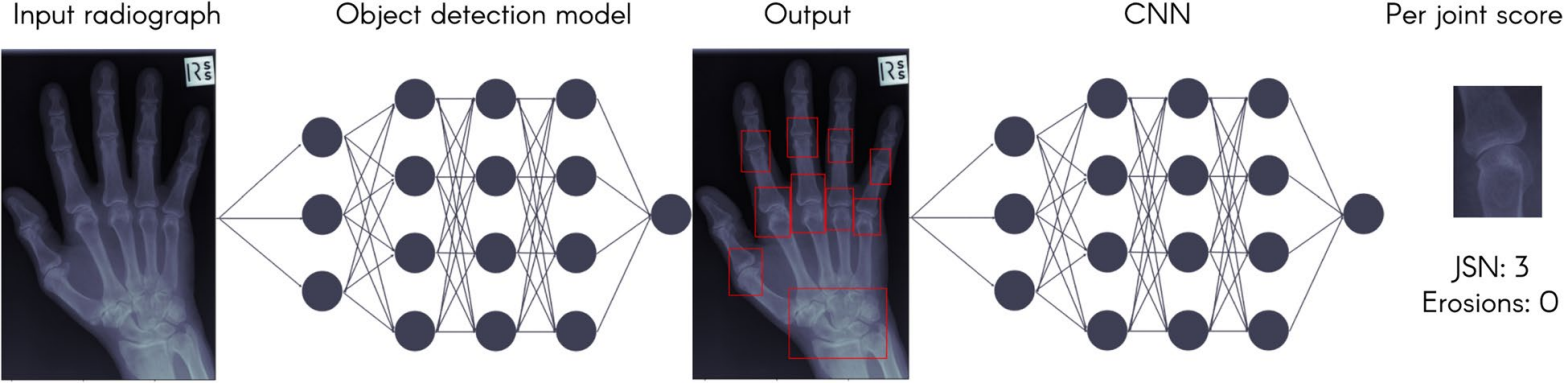
Using a convolutional neural network (CNN), joints are identified from the input radiograph and the score for each joint is assigned

All studies included (*n*=8) were published between 2017 and 2020. Five studies used data from Japan, one used data from Austria, one from Taiwan, and one from Switzerland. Sample sizes ranged from 45 to 5191 radiographs. All studies used trained experts for the baseline scores, usually radiologists or rheumatologists. The majority (6 out of 8) of studies used the SvdH score, reporting erosions, JSN, or both. One paper investigated the presence or absence of erosions while another used the Ratingen scoring method to assess the extent of erosive disease. There were a range of approaches used for joint detection such as histogram of gradients (HOG) [33], multiscale gradient vector flow (MSGVF) [34], and cascade classifiers using Haar-like features [35]. These are all hard-coded methods used to detect or track edges in order to identify structures. Alternate methods used were manual identification of joints or using CNNs. All papers used CNNs in evaluating erosion and narrowing scores. Figure 3 demonstrates this approach of first identifying the relevant joints and then assigning each joint a score.

### Results of individual studies

The first attempt to automate the SvdH score was in 2017 by Morita et al., trained using the hand radiographs of 45 patients with mild to severe RA [24]. Testing was done using leave-one-out cross-validation, with an accuracy of 50.9% and an absolute error of 0.59 for erosions and an accuracy of 64.3% and an absolute error of 0.43 for JSN. They built on this work in 2018 showing modest performance improvements with more data and a different regression technique [25].

In 2018, Murakami et al. used the “Multi Scale Gradient Vector Flow Snakes” method to segment the phalanges of 129 hand radiographs before training with a convolutional neural network to identify the presence or absence of erosions. They achieved a sensitivity of 0.805 and a specificity of 0.99 in a test set of 31 radiographs [26]. A subsequent paper in 2019 built on this work to grade erosions into 6 classes using the Ratingen scoring system [27]. They trained a VGG16 model (a commonly used CNN architecture at the time) using 277 radiographs. In a test cohort of 30 radiographs, they report a sensitivity of 0.924 and a specificity of 0.758. In the same year, Hirano et al. trained on 186 hand radiographs to score the extent of erosions and JSN according to the SvdH scoring system. Joints were first manually clipped and then consensus scored by two rheumatologists. In a test set of 30 radiographs, the JSN sensitivity was 0.880 and specificity 0.748, while for erosion sensitivity was 0.424 and specificity 0.894 [28]. An abstract by Izumi et al. scored erosions based on the difference between two time points using a CNN [31]. With 5-fold cross-validation on a set of 104 radiographs, they reported a mean error of 0.412 SvdH points per joint.

Two abstracts were published in 2020 that used more sizable training sets to score JSN. Deimel et al. used a training set of 3357 radiographs and a test dataset consisting of 1834 radiographs. In the MCPs, their scoring model achieved a sensitivity of 0.844 and a specificity of 0.909, while for PIPs, a sensitivity of 0.863 and a specificity of 0.870 were achieved [29]. In the second abstract, Huang et al. used a dataset of 1397 radiographs, split 70% in training and 30% in testing [30]. While all other studies had focused on only metacarpophalangeal (MCP) and proximal interphalangeal (PIP) joints, they also graded carpometacarpal joints (CMC) and the intercarpal joints in their model, although this was associated with a drop in performance. They had highly reliable baseline scores as they used the consensus of three rheumatologists. Using the cutoff of a score greater than or equal to 2, they found an overall sensitivity of 0.808 and a specificity of 0.919.

### Synthesis of evidence

Preliminary steps have been made in the automation of radiographic scoring, demonstrating the feasibility of this approach. This is largely proof-of-concept work as the majority of papers use datasets that are too small to reach adequate performance. The models have all been tested in data from the same patient cohort and would likely see a drop in performance when used in other contexts. All work thus far has not yet included all joints required in the SvdH score. Despite the recent nature of these studies, many used out-of-date (i.e., non-neural) approaches to joint detection. Neural networks have been shown to consistently outperform such non-neural approaches [36].

Outcome reporting among studies was heterogeneous, making quantitative summary and comparison infeasible. While most studies reported sensitivity and specificity, none reported the area under the receiver operating curve (AUROC)—a key performance metric for diagnostic or prognostic studies [37]. AI-specific guidelines are currently being produced regarding standardized reporting in diagnostic or prognostic studies [38, 39]. Adhering to such guidelines will hopefully foster transparent and consistent reporting of performance metrics and hence allow meta-analyses among studies to be conducted.

Currently, while deep learning has potential in RA, it remains divorced from clinical application. As will be discussed in detail below, larger datasets, the use of newer and more powerful algorithmic techniques, careful evaluation, and standardized reporting of results would all improve the potential for AI systems in radiographic scoring.

## The future of radiographic scoring

Algorithmic scoring shows promise to advance the state of radiographic evaluation by being faster and more sensitive to subtle disease and in mitigating the challenges of low interobserver agreement between scorers. Although the research does not yet adequately test performance to be able to be used in practice, with carefully constructed large datasets and thorough external validation, these advantages could ultimately improve the efficacy and timeliness of pharmaceutical research.

### Efficiency

The most obvious benefit of automated scoring is efficiency. Manual scoring is laborious and requires trained practitioners, taking on average 25 min for a set of seven radiographs from one patient [40]. Instead, an AI system—once trained—would likely take a trivial amount of time to set up, and subsequently would be able to process numerous radiographs per hour [7]. This feature could allow radiographic scoring to be used in the clinic to aid in clinical decision-making (discussed below).

### Improving sensitivity

Effective treatment has resulted in increasingly subtle radiographic findings [41], making the SvdH score less suitable for the spectrum of disease that is seen in current clinical practice. While radiographs are not the most sensitive imaging modality, the score is less sensitive still and can often miss features of joint destruction in order to allow for a reliable and consistent approach [42]. For example, only posteroanterior films are assessed, many of the intercarpal joints are excluded, and other radiographic findings of disease such as soft tissue swelling or juxta-articular osteoporosis are ignored, findings which are known to often predate overt joint destruction in the disease process [15].

A key insight here is that the current approach of predicting human-derived scores may be an unnecessary intermediate step. Deep learning on radiographs can be directly trained to predict relevant outcomes such as functional scores and pain scores, bypassing a human-derived radiographic score completely. By doing so, deep learning methods can learn to detect imaging features that are most predictive of outcomes and make use of other radiographic views, additional joints, and previously excluded imaging findings. Of course, it is possible that a model could learn to identify features related to function that are not caused by RA, most problematically osteoarthritis. Conversely, comorbid fibromyalgia, which is common in long-standing RA, could result in increased pain and decreased function without any anatomical correlates on plain radiography. Carefully designed algorithms will need to account for these confounding factors.

While it is likely that MRI and ultrasound will be increasingly used in pharmaceutical trials given their sensitivity in early disease, the same limitations exist for these imaging modalities regarding manual scores as are apparent for radiographs [43]. Although we discuss radiographs as the current standard outcome measure in this article, similar ideas of improving scoring sensitivity and efficiency can be readily applied to other imaging modalities. Deep learning models could learn to recognize the subtle, early changes of disease detected by any imaging modalities and, in doing so, increase the sensitivity of scoring in RA, whether using radiographs, ultrasound, or MRI.

### Interobserver agreement

The predominant method of assessing the reliability of a scoring system is a measure of interobserver agreement. Even when a system is clearly defined, there is a degree of measurement error [44]. Not only does interpretation differ between people, but an individual’s application of the score can change over time as well. A systematic review of radiographic scoring found that the SvdH score has an intra-rater intraclass correlation coefficient (ICC) of 0.96–0.99 and an inter-rater ICC of 0.90 [42]. Conversely, a deep learning-based system could produce consistent interpretations of similar images, if trained on a sufficiently large and diverse dataset.

### Drug development

The ongoing development of effective treatments in RA is currently restricted by the cost of drug trials [41] and a significant reduction in the extent of joint damage seen on radiographs in the last decade or so [45]. As Landewe et al. highlight, the “signal of progression in the control arm of the trial becomes too low in relation to the unchanged level of noise” and “the beneficial effect of the new therapy can no longer be statistically supported” [41].

A 2020 review [7] showed that in the 15 industry-sponsored drug trials published since 2010, the average baseline SvdH scores ranged from 5.2 to 68.3. Many trials also selected patients who had failed methotrexate treatment and/or had a longer disease duration. While this is an acceptable approach to improve the power of a trial, continuing to recruit patients with severe, treatment-resistant, and long-standing disease limits the external validity of such research to the broader group of patients diagnosed with RA. In particular, these trial results carry little weight in early RA where the so-called window of opportunity invites the study of drugs to prevent radiographic progression from the very earliest stages of disease [46, 47]. The average changes in SvdH score after 1 year ranged from 0.1 to 2.2 in control groups, and the few trials that did report the smallest detectable difference found that it was greater than the average change noted [7]. This makes it difficult to discern whether a score change is due to disease progression or measurement error and thus more challenging to conduct adequately powered clinical trials in RA using current scoring methods.

While it has been argued that subtle differences are not clinically relevant [48], it remains plausible that some patterns of subtle disease are associated with disease progression, and identifying these patients to provide early therapy could prevent the development of clinically significant disease. Furthermore, a major limitation of manual grading is the difficulty in comparing the results of independent trials [49]. An automated system would allow meta-analyses to be conducted more easily in order to provide stronger evidence for the superiority or inferiority of drugs or drug combinations.

### Clinical decision-making

Attempts thus far to bring scoring into the clinical domain have been thwarted by the labor-intensive nature of scoring. The additional information provided by scores could be invaluable to the treating rheumatologist. Automated scoring in the clinical setting could confirm to a reluctant patient that there is evidence of progression that would support a decision to escalate treatment. Conversely, the availability of automated scores could reassure the rheumatologist and patient that the disease is well-controlled and other factors contributing to the patient’s symptoms or experience may need addressing.

Given the heterogeneous nature of RA, being able to more precisely characterize a patient’s disease is particularly useful. We are currently stuck with a trial-and-error approach to management due to the scarcity of validated biomarkers [50]. In addition to any future molecular and genetic biomarkers, imaging biomarkers may also provide information to enable a personalized approach to treatment. Deep learning-based scoring and its ability to detect novel imaging biomarkers in an agnostic, hypothesis-free framework [51] have the potential to advance the role of precision medicine in the management of RA.

### Barriers to automated scoring

Despite the promise of AI, there remain barriers to the development of automated radiographic scoring. The limited availability of large volumes of high-quality training data has hampered the development of AI-based automation. The labeling of training data required is a substantial undertaking given the volume of data and the multiple expert clinical scorers required.

To develop a high-performing scoring model, large volumes of data are required (likely in the realm of thousands of radiographs), drawn from a diverse enough sampling frame that the model can learn the different appearances of joint destruction as imaged with different machines, among differing demographics. Ultimately, for this to be achieved, it is likely that data will need to be pooled across many studies and institutions. The National Consortium of Intelligent Medical Imaging (NCIMI) is an example of efforts to advance AI research in the UK by providing large-scale, de-identified imaging data from multiple sites [52]. Such initiatives are integral to accessing sufficient volumes and variation of training data to develop robust and accurate models.

The evaluation of AI models to score RA radiographs has been limited due to a lack of external validation, with test datasets being from single hospitals or clinics. External validation is crucial in order to be confident that models will function as expected in data from different hospitals/health systems, among different patient demographics, and across varying degrees of disease severity [53]. Models can suffer from poor performance in subsets of a population, and mistakes in certain subsets can have differing clinical significance [54]. For example, underperforming at identifying erosions could be problematic as this is a more overt sign of disease progression and often necessitates treatment escalation. Underperforming in certain minority groups, which is regrettably common given biased training datasets [55], could cause greater healthcare disparities in already disadvantaged groups. Ultimately, as with all clinical tools, the safety of implementation is contingent on testing performance when integrated into the clinical pathway to be confident regarding how the intervention will affect patient outcomes.

These factors have seen many medical AI models fail to reach clinical implementation. Where we have insufficient volume and quality of data, and superficial model evaluation, it is unsurprising when a model fails to perform in practice. A key hurdle though for many medical AI solutions is identifying a clinical use case or solving a particular clinical problem [21]. A deep understanding of the relevant clinical domain is vital when providing such solutions to ensure clinical relevance. Fortunately, radiographic scoring in RA is likely uniquely positioned in this respect. The use case and specific task are already narrow and well defined. Scoring also faces particular constraints for which AI is an apt solution—inadequate sensitivity to subtle disease and poor interobserver agreement. By training models to predict functional sores, AI is not simply replacing an existing system but significantly improving upon what exists in ways that are vital for the ongoing development of new RA treatments. The issue of data volume, quality, and rigorous evaluation, while undeniably onerous, is an issue of resources rather than requiring novel or technical solutions.

## Conclusions

In order to continue developing more effective treatments for RA, we may need new approaches to radiographic scoring that can better detect the subtle disease that is more often seen today. Recent advances suggest that deep learning could be a key tool to tackle the issues faced by manual scoring. A more sensitive and reliable system could increase the statistical power of drug trials so that new therapies or drug combinations can be investigated. Current approaches have automated radiographic scores but fail to take advantage of the main benefits of AI-based models. There remains a mismatch between the strengths of AI and the way it is being used to automate tasks in rheumatology. AI is in a strong position to aid in the continual development of highly effective treatments to improve the quality of life of those living with RA.

## Data Availability

Not applicable

## Abbreviations

AI: Artificial intelligence
AUROC: Area under the receiver operating curve
bDMARDs: Biological disease-modifying antirheumatic drugs
CMC: Carpometacarpal
CNN: Convolutional neural network
DMARDs: Disease-modifying antirheumatic drugs
EHR: Electronic health record
HOG: Histogram of gradients
ICC: Intraclass correlation coefficient
IP: Interphalangeal
JSN: Joint space narrowing
MCP: Metacarpophalangeal
MRI: Magnetic resonance imaging
MSGVF: Multiscale gradient vector flow
NCIMI: National Consortium of Intelligent Medical Imaging
PIP: Proximal interphalangeal
RA: Rheumatoid arthritis
ROI: Region of interest
SvdH: Sharp van der Heijde
SVM: Support vector machine
SVR: Support vector regression
VGG: Visual geometry graph
